# The Integration of Proteomics and Metabolomics Data Paving the Way for a Better Understanding of the Mechanisms Underlying Microbial Acquired Drug Resistance

**DOI:** 10.3389/fmed.2022.849838

**Published:** 2022-05-06

**Authors:** Suereta Fortuin, Nelson C. Soares

**Affiliations:** ^1^African Microbiome Institute, General Internal Medicine, Stellenbosch University, Cape Town, South Africa; ^2^College of Pharmacy, University of Sharjah, Sharjah, United Arab Emirates; ^3^Sharjah Institute for Medical Research, University of Sharjah, Sharjah, United Arab Emirates

**Keywords:** proteomics, metabolomics, microbial drug resistance, virulence, pathogenicity

## Abstract

Due to an increase in the overuse of antimicrobials and accelerated incidence of drug resistant pathogens, antimicrobial resistance has become a global health threat. In particular, bacterial antimicrobial resistance, in both hospital and community acquired transmission, have been found to be the leading cause of death due to infectious diseases. Understanding the mechanisms of bacterial drug resistance is of clinical significance irrespective of hospital or community acquired since it plays an important role in the treatment strategy and controlling infectious diseases. Here we highlight the advances in mass spectrometry-based proteomics impact in bacterial proteomics and metabolomics analysis- focus on bacterial drug resistance. Advances in omics technologies over the last few decades now allows multi-omics studies in order to obtain a comprehensive understanding of the biochemical alterations of pathogenic bacteria in the context of antibiotic exposure, identify novel biomarkers to develop new drug targets, develop time-effectively screen for drug susceptibility or resistance using proteomics and metabolomics.

## Introduction

Since its discovery, an increase in the use of antimicrobials have led to an accelerated incidence of pathogenic drug resistance posing a global public health threat (1, 2). Antibiotics are the most effective treatments of pathogenic bacteria but bacterial antimicrobial resistance (AMR) has become of particular concern globally. A global analysis of AMR found that it was the leading cause of death, particularly in countries with under-resourced health systems, including sub-Saharan Africa (3). AMR occurs when the bacterial cell adapts in the presence of one or more antibiotics and this lead to changes in the bacterial cell causing a reduced antibiotic accumulation within the cell and/or result in the modification of the cell through alterations of the antibiotic target site -modification, -enzymatic alteration, or -replacement (4).

AMR is not only restricted to hospital settings, for example in the case of hospital acquired Methicillin-resistant *Staphylococcus aureus* (HA-MRSA), where the over use and long term exposure of antibiotics has led to an increase in transmission, morbidity and mortality (3, 5–8). A number of gram negative pathogens, including *Klebsiella pneumonia, Pseudomonas aeruginosa, Acinetobacter baumanni, Enterobacter spp*, isolated from patients in intensive care units were found to be multi-drug resistant after extensive use of antibiotics during hospitalization (8). In addition, multi-drug resistance in HA- *Escherichia coli* (*E.coli*) causing urinary tract, abdominal, lung, and blood stream infections has also been increasing in the hospital settings (3, 9).

An antibiotic resistant crisis is emerging due to the overuse and inappropriate use of antibiotics (10). Globally, a rise in community acquired drug resistance has been associated with an increase in morbidity and mortality (3, 10). Several of these include community- acquired MRSA, *Streptococcus pneumonia, Streptococcus pyogenes, Neisseria meningitidis*, bacterial gastroenteritis (*Campylobacter jejuni* or *Salmonella spp*) (11). Globally multi-drug resistant *Mycobacterium tuberculosis* (MDR-TB) is responsible for 25% of all deaths caused by antimicrobial resistant infections (12). Poor adherence, inappropriate regiments and treatment failure are the major causes of acquired MDR-TB. Identifying specific gene mutations, through acquired drug resistance, provides insight into the mechanisms of drug resistance. The global increase in the risk of community transmission may be due to slow bacterial conversion and secondary TB transmission within household contacts (13). Understanding the mechanisms of bacterial drug resistance is of clinical significance irrespective of hospital or community acquired since it plays and important role in the treatment strategy and controlling infectious diseases (14).

Until recently time-consuming conventional methods have been used to determine the susceptibility or resistance of bacteria to a particular antibiotic. Isolated bacteria from biological specimens (blood, spinal fluid, biopsies, etc.) would be grown on culture media and if successful and without contamination the antibiotic resistance and/or minimal inhibitory concentration would be determined. For diagnostic purposes the sub-culturing of pathogenic bacteria for antibiotic susceptibility testing is extremely slow, requiring at least 48 h for the results. Rapid identification of drug resistant isolates has a significant impact on the treatment management of patient and containment of community transmission. In the meantime patients receive broad-spectrum antibiotics that could compromise their health outcome and further lead to the selection of antibiotic resistant bacteria.

Over the last two decades potential of using omics technologies have revolutionized clinical research and have been used to control the spread of pathogens through the identification of antibiotic resistant genes, identifying potential antimicrobial targets and develop highly specific antimicrobial agents (15, 16). There is therefore an urgent need to utilize omics technologies to enable quicker diagnostic of drug resistant pathogens and improve antibiotic treatment regimens.

## Advances in Mass Spectrometry-Based Proteomics Impact in Bacterial Proteomics and Metabolomics Analysis- Focus on Drug Resistance All The Way Through

Over the last few decades mass spectrometry-based proteomics and metabolomics platforms have contributed significantly to our understanding of the bacterial physiology (17) and several studies have analyzed the bacterial cells' response to antibiotic stress (18–20).

The proteome is the complete functional set of proteins expressed by the genome in a cell at a particular time point under specific biological conditions. Proteomics involves the application of high performance mass spectrometry for the identification and quantification of the entire set of protein, the proteome, produced in a cell, tissue or organism (21–23). Two approaches exist for perform in-depth analysis of complex protein samples, data dependent acquisition (DDA) and data independent acquisition (DIA) methods. DDA, mainly used in discovery proteomics can identify, characterize and quantify thousands of proteins in a single sample (24). Whilst in DIA methods, a spectral library constructed from DDA methods are used to predetermine the isolation windows that is used to send precursor ions in the isolation window for fragmentation (25). By using high resolution proteomics multiple biochemical systems can concurrently be investigated quantitatively.

The metabolome is the global collection of the metabolites, the chemical entities transformed during metabolism in a biological system. Metabolomics involves a comprehensive analytical profiling analysis of these metabolites by measuring subsets of these small molecules; lipids, free fatty acids, bile acids, sugars, organic acids, amino acids, that have distinct physical properties (26). Metabolomics offer a unique opportunity to combine high-throughput analytical chemistry and multivariate data analysis to characterize of metabolic phenotypes and advance our understanding of the AMR mechanisms and how it correlates with changes in metabolite concentration (27, 28).

The development and technological advances of proteomics and metabolomics technologies offers a unique opportunity to understand these complex mechanisms of specific diseases, e.g., bacterial drug resistance (28–30).

Mass spectrometry based proteomic analysis of *in vitro* cultured bacterial isolates allows the opportunity to quantitatively compare proteomes under various controlled conditions. The first comparative proteomic analyses of susceptible and drug resistant bacteria using 2 dimensional 2D gel electrophoresis-based (2-DE) combined with matrix-assisted laser desorption/ionization time of flight mass spectrometry (MALDI-TOF MS) dates back more than a decade ago (31–36). Valéria dos Santos et al. (34) identified a total of 12 proteins that were more abundant in an *in vitro* derived piperacillin/tazobactam-resistant strain of *E.coli* to be associated with antibiotic resistance and bacterial virulence when compared it is susceptible wild type strain. In a separate proteomics analysis of Wang et al. (37) also found proteins associated with virulence we significantly abundant in a vancomycin-resistant *Enterococcus faecalis* compared when compared to a clinical isolate. Several methods using MALDI-TOF MS are employed in diagnostic laboratories to characterize drug- resistance and susceptibility (38). In recent years label-free quantitative LC-MS/MS have been developed to provide a simplified sample preparation, where the whole proteome is digested into peptides without prior protein separation, to analyse complex proteomic samples and directly identify thousands of proteins quantitatively (Table 1) (29, 39, 41–44). Pournaras et al. (45) in their comparative proteomic analysis of colistin-susceptible and resistant *A. baumannii* strains found proteins involved in antibiotic resistance and virulence were differential expressed in resistant strains. Interestingly, these antibiotics (piperacillin/tazobactam, vancomycin and colistin) target the cell wall and cell membrane. In a separate study, the proteomic analysis of three clinical diarrhoeagenic *E.coli* isolates; two enteropathogenic *E.coli* (EPEC) [one resistant to ciprofloxacin] and one Shiga-toxin-producing *E.coli* (STEC); directly collected from stool samples enabled the identification of antimicrobial resistant mechanisms (43). They found that key virulence proteins (FebB, YbhA, YeiP, Fdx, LolA, YaeT, OmpA) were more abundant in susceptible isolates while it several were undetected in the resistant isolates. Interestingly, the loss of these virulence proteins in the drug resistant isolates did not affect the survival but were associated an with increased resistance to multiple antibiotics and could be responsible for the transmission of resistant strains (43, 46). In addition, the resistant EPEC isolate showed a significantly higher abundance of metallo-beta-lactamase family proteins, and ABC superfamily of efflux pump proteins that are chromosomally encoded and can cause resistance to multiple drugs when overexpressed (43, 47, 48).

**Table 1:** An overview of the methods and results generated in the five studies using mass spectrometry-based proteomics and metabolomics.

**References**	**Strain/species**	**Enrichment**	**Fractionation method**	**# Proteins/metabolites identified**
Suh et al. (39)	*Klebsiella pneumonia*	Global analysis	LC-MS	1,538 proteins
Lin et al. (40) Lin et al. (29)	*E.coli*	Global analysis; Fe^3+^-IMAC Phosphopeptide Enrichment	LC-MS/MS	2,567 proteins and 1,133 phosphorylated proteins
Giddey et al. (41)	*M. smegmatis*	Cell wall enrichment	LC-MS/MS	2,283 proteins
Lin et al. (29)	*E.coli*	Global	GC-MS	273 metabolites
Mielko et al. (28)	*Pseudomonas aeruginosa*	Global	NMR	32 intracellular metabolites

In a separate study, Suh et al. used label-free quantitative LC-MS/MS to perform a global proteome analysis to determine the biochemical alterations that would occur when beta-lactamase producing *Klebsiella pneumonia* are exposed to sub-lethal concentrations of antibiotics (doxycycline and streptomycin). They found that the expression of 97 proteins; 55 and 42; were elevated in when exposed to doxycycline and streptomycin, respectively. Several outer membrane proteins in cells treated with doxycycline were reduced compared to untreated cells. (39). Recently Giddey et al. (41) showed that the cell wall enriched proteome of drug resistant *Mycobacterium smegmatis* mutant exposed to sub-lethal concentration of rifampicin phenotypically adapts independently from the rpoB mutation. The combination of these study and many others has allowed for the construction of a database of proteins involved in the cellular biochemical processes of resistance to drugs (49).

Significant advances in mass spectrometry-based proteomics workflows makes it possible to address questions regarding clinical isolates and their response to antibiotics. Proteomics analysis directly on single colonies isolated from clinical samples enables the exploration of the proteomes of minimally passaged clinical isolates from primary culture plates (50, 51). Fortuin et al. (50) analyzed the proteome of 6 individually isolated single colonies and identified approximately 40% of the theoretical *E.coli* proteome. Several of these proteins were involved in swarming motility, key virulence factors only expressed within the biofilm-like microenvironments of single colonies and would have otherwise been missed in proteomic analysis perform on liquid cultures.

On the contrary, targeted proteomics systematically analyze a set of targeted proteins where the mass spectrometer only acquire fragment ion signals for peptides from these preselected proteins. These kinds of measurements rely on the acquisition mode of the mass spectrometer, i.e., selected reaction monitoring (SRM), multiple reaction monitoring (MRM) and parallel reaction monitoring (PRM). For example, Haag et al. (52) used SRM to demonstrate that it is a time- and cost-effective technique to determine drug susceptibility and resistance of ampicillin and chloramphenicol resistant *E.coli*.

Sequential Windowed Acquisition of All Theoretical Fragment Ion Mass Spectra (SWATH-MS) is a DIA method used to complement DDA MS the MS2 spectra are observed and quantified in a targeted manner according to an established assay spectrum library. Sidjabat et al. applied LC-MS/MS in combination with SWATH to quantitatively identify 724 proteins in 9 *E.coli* isolates that were exposed to meropenem or ciprofloxacin. With their analysis they found that antibiotic exposure affect specific proteins involved in the bacterial cell's adaptation and survival under antibiotic pressure. Similar to previous studies they identified a set of outer membrane proteins that were specifically induced under antibiotic pressure (53).

The emerging field of discovery metabolomics allows for the in-depth investigation of organized cascades of metabolic process using analytical MS methods [nuclear magnetic resonance (NMR) spectroscopy, gas chromatography MS (GC-MS), liquid chromatography MS (LC-MS) and capillary electrophoresis coupled to MS (CE-MS)] (Table 1) (54). Conventional NMR techniques have been used to study antimicrobial peptides despite its limited capacities for analyzing complex samples due to its low sensitivity and the metabolites have to be present in sufficient quantities for detection (55, 56). However, it does provide a broad coverage of metabolites, without prior separation of metabolites, which can be used for screening of global metabolites (15). The development of metabolomics techniques is mainly by the advances in mass spectrometry. Recently, Aries and Cloninger (57, 58) used NMR spectroscopy to identify the metabolic profile of drug resistant *E.coli* and B. cereus and revealed that the concentration of metabolites, particularly those involved in peptidoglycan synthesis, altered when cultured in the presence of antimicrobials.

Meilko et al. used ^1^H NMR to analyze intra- and extracellular metabolites of susceptible MDR *P. aeruginosa* strains. Their results showed that there is a significant difference in the intracellular metabolites involved in amino acid turnover, protein synthesis, protein uptake and the protein biosynthesis and degradation (28). Lin et al. used GC-MS based metabolomics and characterized 273 metabolites in a comparison of two drug sensitive and two multidrug resistant (MDR) clinical *E.coli* strains. The results showed that the metabolite profiles of the two MDR strains were different and the bioinformatic analysis revealed that metabolites involved in carbon metabolism, and pyrimidine metabolism pathways, ABC transporters, and involved in cysteine and methionine metabolism pathways were more enriched compared to the drug sensitive strain (29).

Metabolomics using LC-MS/MS studies can be categorized into targeted and untargeted approaches. Targeted metabolomics involves the preselection of chemically characterized analytes prepared using selective sample preparation optimized for their specific physical-chemical properties of the target compounds followed by chromatography in combination with mass spectrometry (59, 60). Global targeted metabolomics is used to generate a metabolic fingerprint using the overall metabolites identified in a single sample (61). The simultaneous measurement of metabolites in a single sample consequently requires high performance bioinformatics tools to analyse the large complex metabolite datasets (60). Currently there is a limited number of studies that have used the advances of mass spectrometry to investigate the metabolite landscape of bacteria in relation to drug resistance. The most commonly targeted metabolomics method is multiple reaction monitoring (MRM) mode coupled to mass spectrometry and has been the foundation of high-quality metabolite quantitation which can be can be mapped to a metabolic flux involved in antimicrobial resistance pathways (62).

## Need to Integrate Proteomics and Metabolomics data Sets Focused on Drug Resistance

Understanding the drug resistance mechanisms of currently used antibiotics can improve the incorporation of new therapeutics to improve the treatment of current antibiotics (63, 64). Proteomics and metabolomics on their own have previously shown to be powerful platforms that enable us to understand the biochemical alterations of biological systems under specific environmental conditions and triggers. Despite continuous development to increase sensitivity and specificity in detection of these molecules, proteins and small metabolites, there are still limitations to in the interpretation of the data, particularly in the understanding of clinical pathogenic bacteria. When analyzed separately, it is easy to forget the intimate connection between proteins and metabolites. The building blocks of proteins are amino acids, also metabolites, can be catabolised and produce other metabolites, urea, pyruvate and ammonia, etc. which in turn can induce alterations in the metabolome.

Antimicrobial resistant strains, including *P.aeruginosa, E.coli* and *Klebsiella pneumoniae*, have the ability to form biofilms, creating a physical barrier to protect the bacteria from antibiotics (28). Metabolomics analysis helped unravel *P.auruginosa* polymyxin resistance mechanism through correlating the intra and extracellular metabolite concentrations and its association with the surrounding bacterial environment (65).

Zhao et al. (66) applied a multi-omics (genome, proteome and metabolomics) approach, using MetaboAnalyst, to investigate capreomycin (CAP) resistant *Mycobacterium tuberculosis* (*M.tb*) strains. They used LC-MS based metabolomics and labeled proteomics and revealed a new CAP resistance mechanism to tlyA-deficient and mutated *M.tb* strains. The tlyA deficient *M.tb* strains exhibited greater drug tolerance than the tlyA point mutation *M.tb* strain and may be associated with the weakening of SAM-dependent methyltransferase (SDM), AdoMet-MT, activity and have an impact on membrane lipid metabolism. LC-MS based metabolomics and proteomics analysis of 10 clinical susceptible and 10 extended-spectrum b-lactamase producing (resistant) *E.coli* strains allowed to discriminated between the susceptible and resistant strains. Furthermore, the correlation between the differentially abundant proteins and metabolites revealed that specific metabolites in purine metabolism pathway may play an important role in antibiotic resistance. These molecular targets (proteins and metabolites) might be important modulators of may be effective for treatment of drug resistant strains (30). The minimum inhibitory concentration (MIC) test used to measure the lowest effective concentration of an antimicrobial agent, under defined conditions, that inhibits visible growth of a bacteria is time consuming and rely on culture dependent methods. Proteomics and metabolomics can be used to identify molecules in clinical isolates targeted by antibiotics that cause antibiotic resistance. Specifically, in the case of clinical isolates exposed to antibiotics that bind to target proteins causing alterations in protein abundance, enzymatic activity, mechanism of drug action or cause protein modifications will be able to provide insight into pathways that result in antibiotic resistance (54, 67). In combination, the MIC and protein and metabolite targets can for used in prescribing appropriate treatment and monitoring infection patterns (68).

## Recommendations for Future Research

Liquid chromatography in combination with mass spectrometry, LC-MS, is the analytical foundation of proteomics and metabolomics. The integration of the proteome and metabolomic has the potential to complement and correlate data that will provide insight on the dynamic drug resistance mechanisms. The advances in LC-MS have revolutionized field of proteomics and metabolomics analyses, particularly our understanding of bacterial physiology and drug resistance. The development of LC-MS tools to analyze single cell proteomics and metabolomics will be extremely useful in the field of clinically relevant AMR and provide the tools to perform omics studies to globally and simultaneously measure different molecules present in a biological sample. Proteins and metabolites are the end products of genomic and transcriptomic and pathway alterations that underlie physiological homeostasis and pathogenesis.

Comprehensive proteomic and metabolomic reference databases is a crucial part of efficient proteomic and metabolomic analysis (69). Until recently metabolite identification was restricted to the human metabolome database (70). It should be encouraged to make proteomics and metabolomics raw data sets publicly available platforms such as Proteome Identification Database (PRIDE) and Metabolomics workbench upon publication so that it can be used to create more comprehensive databases that include reference databases for all organisms.

Over the last few years, several bioinformatics tools have been developed to allow multi-omics data integration. These include, MetaboAnalyst, Paintomics 3.0, Burrito, Mimosa and OnPLS multi-bock modeling (Table 2) and can be used separately and in combination (71, 72, 74–83). The use of multi-omics data integration in AMR provide an opportunity to expand a multi-level analysis in making drug target predictions, -responses and -prognostic biomarkers for easier and quicker diagnosis and treatment options.

**Table 2:** Summary of -omic data integration analysis tools.

**References**	**Analysis tool**	**Types of data**	**Features**
Chong et al. (71); Pang et al. (72); Xia et al. (73–75)	MetaboAnalyst	- Transcriptomics - Proteomics - Metabolomics	- Web based - Network explorer feature to integrate -omics datasets - Integrate -omics data for joint pathway and network analysis
Chong et al. (76); Chong and Xia (77); Pang et al. (78)	MetaboAnalystR	- Genomics - Transcriptomics - Proteomics - Metabolomics	- R-package - Raw spectra processing - Global metabolomics analysis - Metabolite-Gene interactions - Batch analysis
Srivastava et al. (79)	OnPLS multi-bock modeling	- Transcriptomics - Proteomics - Metabolomics	- Symmetrical multi-block method that does not depend on the order of analysis when more than two blocks are analyzed.
García-Alcalde et al. (80); Hernández-de-Diego et al. (81)	Paintomics 3.0	- Transcriptomics - Proteomics - Metabolomics	- Web based tool - Joint visualization of transcriptomics proteomics and metabolomics data
McNally et al. (82)	Burrito	- Genomics - Metabolomics	- Web-based - Visualization tool - Taxonomic-to-phenotyping - Relationship between taxonomic and functional abundances across microbiome samples
Noecker et al. (83)	MIMOSA2	- Genomics - Metabolomics	- R- and web based - Metabolic network-based tool for inferring mechanism- supported relationships in microbiome-metabolome data

**Figure 1:**
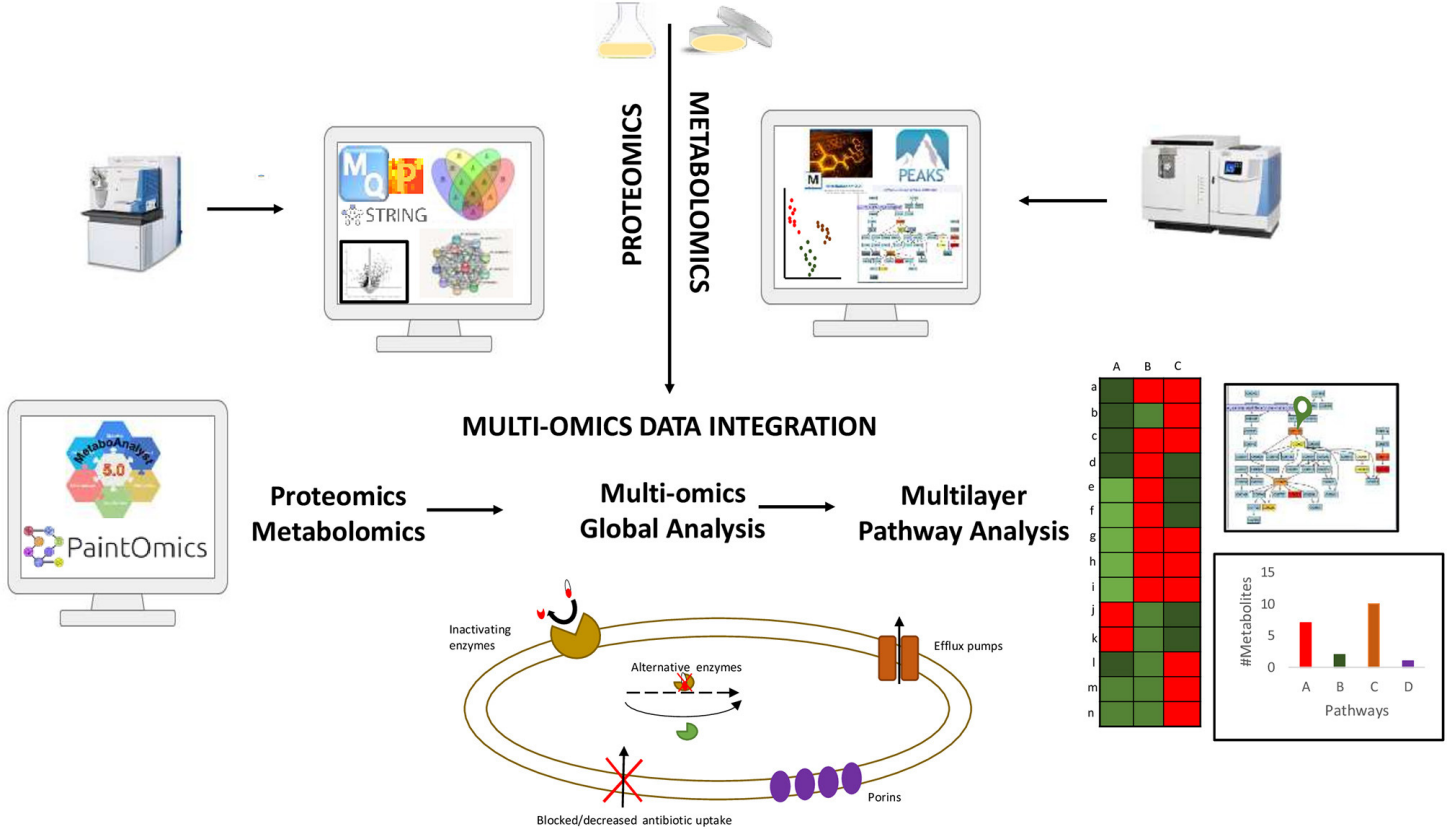
Summary of data integration workflow combining proteomics and metabolomics data for a comprehensive understanding of the biochemical alterations of pathogenic drug resistant bacteria.

The capacity now exists to incorporate multi-omics studies in order to obtain a comprehensive understanding of the biochemical alterations of pathogenic bacteria in the context of antibiotic exposure, identify novel biomarkers to develop new drug targets (Figure 1). Thus, the eventual incorporation and translation of omics approaches into medical microbiology practice would, first and foremost, improve long-standing antimicrobial tests/assays, resulting in more accurate, time-effective, and informative diagnosis, and, eventually, drug and therapy monitoring.

## Data Availability Statement

The original contributions presented in the study are included in the article/supplementary material, further inquiries can be directed to the corresponding authors.

## Author Contributions

SF and NS contributed for the conceptualization and writing of the manuscript. All authors contributed to the article and approved the submitted version.

## Conflict of Interest

The authors declare that the research was conducted in the absence of any commercial or financial relationships that could be construed as a potential conflict of interest.

## Publisher's Note

All claims expressed in this article are solely those of the authors and do not necessarily represent those of their affiliated organizations, or those of the publisher, the editors and the reviewers. Any product that may be evaluated in this article, or claim that may be made by its manufacturer, is not guaranteed or endorsed by the publisher.
